# Pro-Inflammatory Mediation of Myoblast Proliferation

**DOI:** 10.1371/journal.pone.0092363

**Published:** 2014-03-19

**Authors:** Jeffrey S. Otis, Sarah Niccoli, Nicole Hawdon, Jessica L. Sarvas, Melinda A. Frye, Adam J. Chicco, Simon J. Lees

**Affiliations:** 1 Medical Sciences Division, Northern Ontario School of Medicine, Thunder Bay, Ontario, Canada; 2 Department of Biology, Lakehead University, Thunder Bay, Ontario, Canada; 3 Department of Health and Exercise Science, Colorado State University, Fort Collins, Colorado, United States of America; 4 Department of Biomedical Sciences, Colorado State University, Fort Collins, Colorado, United States of America; 5 Department of Kinesiology and Health, Georgia State University, Atlanta, Georgia, United States of America; Universidad Pablo de Olavide, Centro Andaluz de Biología del Desarrollo-CSIC, Spain

## Abstract

Skeletal muscle satellite cell function is largely dictated by the surrounding environment following injury. Immune cell infiltration dominates the extracellular space in the injured area, resulting in increased cytokine concentrations. While increased pro-inflammatory cytokine expression has been previously established in the first 3 days following injury, less is known about the time course of cytokine expression and the specific mechanisms of cytokine induced myoblast function. Therefore, the expression of IL-1β and IL-6 at several time points following injury, and their effects on myoblast proliferation, were examined. In order to do this, skeletal muscle was injured using barium chloride in mice and tissue was collected 1, 5, 10, and 28 days following injury. Mechanisms of cytokine induced proliferation were determined in cell culture using both primary and C2C12 myoblasts. It was found that there is a ∼20-fold increase in IL-1β (p≤0.05) and IL-6 (p = 0.06) expression 5 days following injury. IL-1β increased proliferation of both primary and C2C12 cells ∼25%. IL-1β stimulation also resulted in increased NF-κB activity, likely contributing to the increased proliferation. These data demonstrate for the first time that IL-1β alone can increase the mitogenic activity of primary skeletal muscle satellite cells and offer insight into the mechanisms dictating satellite cell function following injury.

## Introduction

Skeletal muscle is a highly adaptable tissue and capable of robust regeneration in response to injury. In skeletal muscle, the resident stem cells responsible for tissue repair are termed satellite cells (SC). In intact skeletal muscle, SCs exist as a quiescent population of cells located between the basal lamina and plasmalemma [1]. When exposed to signals from damaged tissue, satellite cells exit the quiescent stage and enter the cell cycle [2], [3]. These proliferating satellite cells become a population of muscle precursor cells (MPCs), which play a key role in regeneration [4]–[7].

While the mechanism of injury can vary (e.g., strenuous physical activity, contusions, genetic defects, or exposure to toxins), the response involves a similar coordinated series of events leading to repair. Injuries result in necrosis of damaged tissue, which induces a rapid and sequential inflammatory response. Neutrophils are the first to infiltrate the damaged tissue, and increase in number within 1 hour post injury [8]. Following neutrophil infiltration, macrophages begin to populate the injury site. These macrophages remove cellular debris from the area by phagocytosis, prevent muscle cell apoptosis, and secrete various cytokines and growth factors to facilitate muscle fiber repair. The early inflammatory cascade involves mainly pro-inflammatory macrophages (M1) that secrete several pro-inflammatory cytokines such as tumour necrosis factor-α (TNF-α), interleukin-1β (IL-1β), and interleukin-6 (IL-6) [9]–[13]. The secretion of TNF-α, IL-1β, and IL-6 by M1 macrophages during the pro-inflammatory stage coincides with MPC proliferation [14].

A strong link exists between inflammatory cell function and skeletal muscle repair. For example, blocking or minimizing the impact of inflammatory cells using non-steroidal anti-inflammatory drugs (NSAID) impaired muscle regeneration [15], [16], weakened musculotendinous units [17], [18], and diminished SC function [19], [20] compared to untreated animals. In addition, macrophage depletion causes increased fibrosis and impaired regeneration of skeletal muscle post-injury [21]. However, less is known about the coordinated immune response leading to increased cytokine expression in the regulation of MPCs and their contribution to skeletal muscle repair.

IL-1β has pleiotropic effects in many cell types [22]. Following skeletal muscle injury, elevated tissue IL-1β expression is observed within 2 days and likely reflects release from T cells and macrophages [23], [24]. This expression has been linked to a decrease in muscle specific proteins and myotube size [25] and has been shown to have mitogenic effects in human smooth muscle cells when acting together with IL-1α [26]. IL-1β has also been shown to increase TNF synthesis in macrophages [27] and IL-6 expression in neuroendocrine cells [28]. IL-1β expression is known to initiate expression of mitogens in fibroblasts that will stimulate proliferation of mesenchymal cells in a paracrine manner [29], and when human myoblasts were exposed to activated monocyte conditioned media, proliferation and IL-6 expression were both increased [30].

In particular, it is known that endogenous expression of IL-6 has mitogenic effects on MPCs [31], [32]. MPCs isolated from IL-6^−/−^ mice exhibit reduced proliferation, which can be rescued with the addition of recombinant IL-6 [32]. Furthermore, in mice lacking IL-6, there was reduced macrophage recruitment to the injury site, along with decreased MPC proliferation [33]. This suggests that IL-6 secretion by both MPCs and M1 macrophages facilitates the regeneration process. Both IL-1β and TNF-α have been shown to increase IL-6 expression and release, as well as reduce myogenic differentiation [28], [34]–[36]. However, the mechanism of IL-1β induced IL-6 expression, and the relationship between TNF-α, IL-1β, and IL-6 during the critical stages of MPC proliferation have yet to be resolved. The purpose of the present study was to determine the effects of IL-1β on MPC proliferation, and to gain further insight into the role of the IL-1β/IL-6/TNF-α axis. Results from this study have shown for the first time the timeline of IL-1β up regulation following skeletal muscle injury. In addition, the elevated IL-1β does increase proliferation of MPCs and myoblasts, and is likely accomplishing this through activation of NF-κB. Therefore, the transient increase in IL-1β following injury is significant, coinciding with the activation and proliferation of MPCs, which is a required step leading to the terminal differentiation of MPCs resulting in *de novo* muscle fiber formation.

## Methods

### Animals

Fisher 344 x Brown Norway F1 hybrid, male rats were obtained from the National Institute on Aging. Male C57BL/6 mice (∼25 g) were purchased from Jackson Labs (Bar Harbor, ME). In order to minimize suffering, animals were anesthetized by intraperitoneal injection of a xylazine (15 mg/kg) and ketamine (100 mg/kg) cocktail. Animals were housed at 21°C on a 12-hr light/12-hr dark cycle and allowed free access to food and water. For tissue collection, animals were given an intraperitoneal injection of ketamine (80 mg/kg), xylazine (10 mg/kg), and acepromazine (4 mg/kg). The animals were killed by removing the heart, and the tissue was excised.

### Ethics Statement

All procedures that involved rats were approved by the Institutional Animal Care and Use Committees at Colorado State University (protocol# 11-2947A) and Lakehead University (protocol# 13-2011). All procedures that involved skeletal muscle injury in mice were approved by the Institutional Animal Care and Use Committee at Emory University (protocol# 069-2010).

### Cell Culture

Primary MPCs were isolated as described previously [37]–[44]. Briefly, cells were isolated from the gastrocnemius and plantaris muscles by pronase digestion and pre-plated for 24 hours on tissue-culture treated 150-mm plates. MPCs were cultured on Matrigel (BD Biosciences, San Jose, CA) coated plates (0.1 mg/ml Matrigel, 60 minutes at 37°C) and passaged only one time (growth media (GM), 20% FBS in Ham’s F-10; 6% O_2_, 5% CO_2_, and 89% N_2_ at 37°C). C2C12 mouse myoblast cells were obtained from American Type Culture Collection and maintained 5% CO_2_ 37°C 10% FBS in Dulbecco’s Modified Eagle’s Medium supplemented with penicillin (100 U/ml) and streptomycin (100 μg/ml). Cytokines were added to the cell culture media at the concentration indicated. For experiments involving soluble tumour necrosis factor receptor I (sTNFRI), the cytokines and sTNFRI (0.3 μg/ml) were pre-incubated for 2 hours at 37°C in cell culture media prior to treatment. For experiments to inhibit NF-κB, ammonium pyrrolidine dithiocarbamate (PDTC, Sigma, St. Louis, MO) was used at a concentration of 50 μM and cells were pretreated for 1.5 hours at 37°C prior to cytokine addition.

### Barium Chloride-induced Skeletal Muscle Injury

Fifty-μl of a 1.2% barium chloride (BaCl_2_) solution (diluted in sterile saline) was injected into mouse TA muscles using a 27 G needle as previously described [45]. Contralateral TA muscles served as uninjured controls. Briefly, the needle was inserted at the origin of the TA, extended past the mid-belly of the muscle to a region just superior to the distal tendon. Next, the diluted BaCl_2_ solution was continuously injected into the TA as the syringe was removed. Complete serial sections of the injured TA confirmed that the mid-belly section was affected by the myotoxin. Injured and uninjured, contralateral control muscles were harvested *post mortem* at 1, 5, 10, and 28 days following injury and processed for markers of degeneration and regeneration.

### Histological Assessment of Regeneration in Injured TA Muscles

Uninjured and injured TA muscles were removed, embedded in OCT, and immediately frozen in isopentane cooled in liquid nitrogen. To ensure that we were assessing the most damaged portion of the TA, muscles were cut in serial sections at 10 μm beginning at the mid-belly. Sections were then processed for hematoxylin and eosin staining, dehydrated, mounted, and visualized at 10X with a Leica light microscope as previously described [46]–[49]. Cross-sectional areas of approximately 200 centrally-nucleated fibers (i.e., regenerated fibers) per muscle were calculated using ImageJ software (NIH, Bethesda, MD).

### Real-time Polymerase Chain Reaction (RT-PCR)

Injured and contralateral control TA muscles were immediately frozen in liquid nitrogen and stored at −80°C until processed for RT-PCR analyses as previously described [46], [48], [50]. Briefly, trizol was added (1 ml/100 mg tissue) and the tissues homogenized using an electric tissue homogenizer. Total RNA (2.5 μg) was reverse transcribed in a 25–50 μl final reaction volume using random primers and M-MLV reverse transcriptase (Invitrogen, Carlsbad, CA). The reverse transcription reaction was incubated at 65°C for 10 min, 80°C for 3 min, and 42°C for 60 min. RT-PCR products were analyzed using the iCycler iQ system (Bio-Rad, Hercules, CA). cDNA (5 μl of a 1∶10 dilution) was amplified in a 12.5 μl reaction containing 400-nm gene-specific primer pair and iQ Sybr Green Supermix (Bio-Rad). Primers were as follows: IL-1β, 5′-AGAGCATCCAGCTTCAAATCTC-3′ and 5′-CAGTTGTCTAATGGGAACGTCA-3′; IL-6, 5′-CAAAGCCAGAGTCCTTCAGAG-3′ and 5′-GTCTTGGTCCTTAGCCACTCC-3′. As a control, RT-PCR was also performed on 2 μl of each RNA sample to confirm absence of contaminating genomic DNA. For primary MPC mRNA measurements, samples were lysed at 2, 4, and 24 hours post-stimulation with IL-1β in RLT-lysis buffer with 1% β- mercaptoethanol and passed through a QIAshredder (Qiagen, Valencia, CA). RNA purification was performed with the on-column DNase I digestion using the RNeasy micro kit (Qiagen) according to the manufacturer’s instructions. RNA was quantified spectrophotometrically and purity was assessed by measuring the ratio of the absorbance at 260 nm and 280 nm, and RNA integrity was verified using agarose electrophoresis. RNA was reverse transcribed using SuperScriptTM III first-strand cDNA synthesis system (Invitrogen) with random hexamer primers. RT-PCR was performed using Sybr Green Master Mix and an ABI Prism 7000 (Applied Biosystems, Foster City, CA). The specificity of the primer pair was evaluated using agarose gel electrophoresis; only a single product of appropriate size was observed. 25 ng of cDNA for each sample was used. Standard curves for all targets and 18S rRNA were run to determine amplification efficiency. All reactions were performed in duplicate and the starting quantity of the gene of interest was normalized to 18S rRNA for each sample. Data are represented as means ± range of potential values based on the 2^−ΔΔCT^ method with the error expressed as the expected low (2^(−ΔΔCT+SD)^) and expected high (2^(−ΔΔCT − SD)^) [46]–[49], [51] and expressed as fold changes relative to uninjured controls.

### Proliferation

To analyze cell proliferation, 5-bromo-2′-deoxyuridine (BrdU) incorporation was determined using flow cytometry as described previously [37], [39], [42]. For MPC and C2C12 proliferation, cells were plated in GM and cultured for 24 hours. After 24 hours, cells were either treated with cytokine, pre-incubated cytokine and sTNFRI, or vehicle. In experiments to inhibit NF-κB, cells were pretreated for 1.5 hours with 50 μM PDTC prior to cytokine addition. The cells were then pulsed with BrdU for 60 min beginning either 23 hours following treatment, or 20 hours following treatment during NF-κB inhibition. 20,000 cells were analyzed using a FACS-Calibur flow cytometer and CellQuest Pro (BD Biosciences, San Jose, CA).

### Luciferase

Transient transfections were performed using Fugene 6 (Roche Applied Science, Indianapolis, IN), following the manufacturer’s instructions. Cells were transfected with the nuclear factor-kappa B (NF-κB) cis-reporter construct, which contains 5 repeats of the transcription recognition sequence (TGGGGACTTTCCGC) linked to a basic promoter element (TATA box) and the firefly luciferase gene (Stratagene, La Jolla, CA). The pRL-CMV Renilla luciferase reporter vector (Promega, Madison, WI) was co-transfected in each experiment and used as an internal control promoter in order to normalize for transfection efficiency. A total of 1 μg of DNA for each well on a 6-well plate was used for both firefly and Renilla luciferase reporter constructs at a firefly:*Renilla* ratio of 20∶1. Cells were lysed using passive lysis buffer (Promega, Madison, WI) and stored at −80°C. Firefly and *Renilla* luminescence were measured using the Dual-Luciferase Reporter Assay System (Promega) on a FLUOstar microplate luminometer (BMG Labtech, Ortenberg, Germany).

### Statistics

Data are presented as mean ± SEM. Sample sizes are indicated for each measurement in the figure legends. Comparisons between groups were done using ANOVA and the Fisher’s LSD post-hoc analysis (SigmaStat software, Systat, Chicago, IL). Significance was accepted at p≤0.05.

## Results

**Figure 1:**
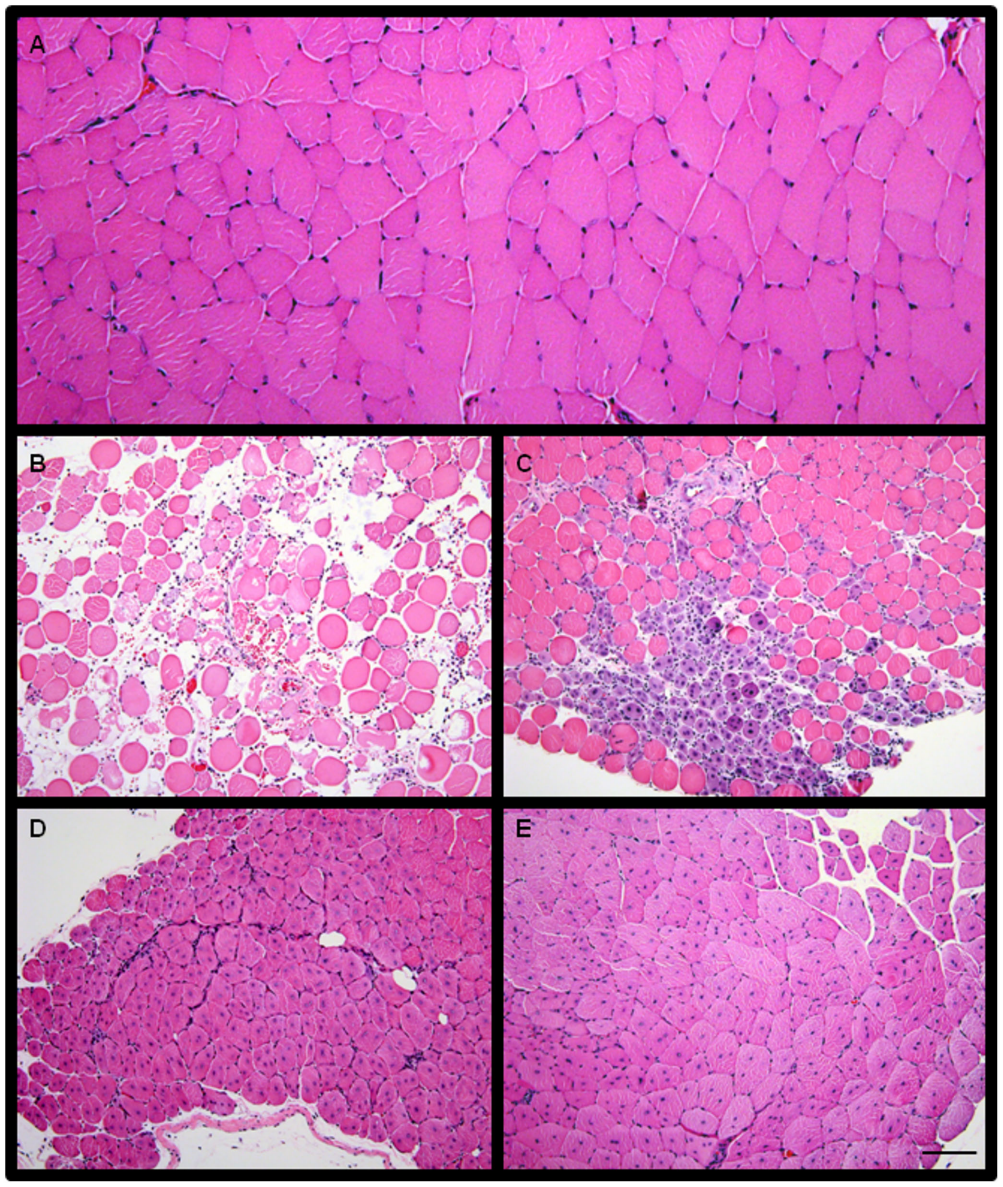
Histological representation of the progression of injured muscle through repair and regeneration. (A) Uninjured tibialis anterior (TA) muscle. Injured TA muscle (B) 1 days, (C) 5 days, (D) 10 days, and (E) 28 days after barium chloride (BaCl_2_) injection. Bar in E = 100 μm. Images taken at 10X magnification.

Immediately following injury, an immune response is triggered that assists the muscle in progressing through reparative phases. Specifically, an increase in pro-inflammatory cytokine content is observed within the first 2–3 days following injury (reviewed by Tidball et al. [52]). However, very little is known about the expression level of IL-1β and IL-6 over several weeks following skeletal muscle injury that contributes to complete regeneration. In order to better understand the time course of pro-inflammatory cytokines, we injured skeletal muscle and collected tissues at 1, 5, 10, and 28 days following injury (Figure 1). These time points were selected because they reflect unique and distinct stages of skeletal muscle regeneration ranging from early inflammatory events (day 1), initial regeneration and regrowth (days 5 and 10), and complete recovery (day 28) [45], [53]–[58]. As seen in Figure 1B, 1 day following injury, the normal skeletal muscle structure has been disrupted and increased oedema and cells in the interstitial space are observed. While there are still increased cells in the interstitial space at 5 days following injury, small muscle fibers with centrally located nuclei are observed (Figure 1C). 10 days following injury, the newly forming fibers are larger (Figure 1D) and by 28 days the regenerating fibers are approaching the size of controls (Figure 1A and E).

**Figure 2:**
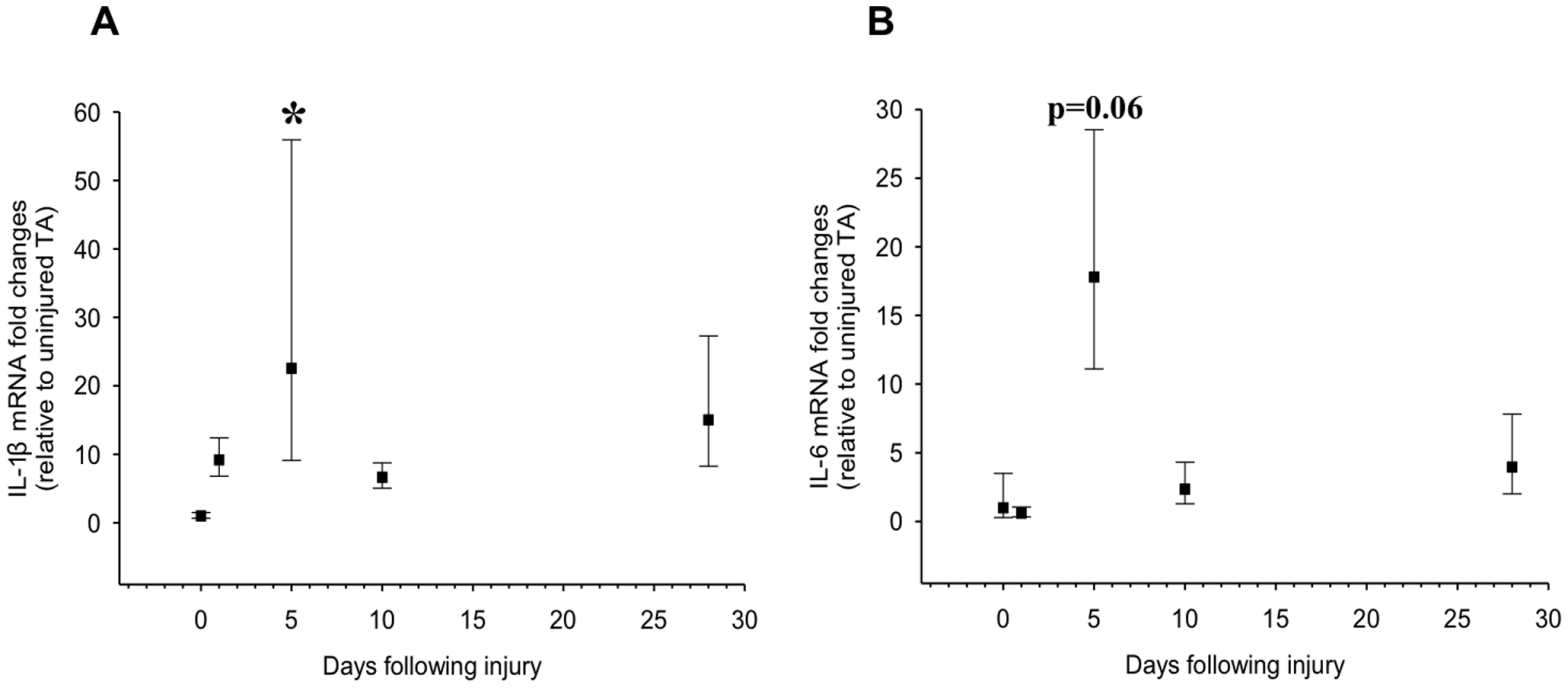
IL-1β and IL-6 mRNA expression post-injury to the tibialis anterior (TA). TA muscle was injured using a barium chloride injection method and muscles were collected 1, 5, 10, and 28 days post-injury and analyzed for (A) IL-1β and (B) IL-6 mRNA expression levels. Data are expressed as fold-increase +/− expected high and low expression relative to the average value [51]. *denotes significance (p≤0.05) compared to uninjured control (n = 3–4 per time point).

mRNA expression levels of IL-6 and IL-1β at multiple time points following injury to the tibialis anterior muscle were measured (Figure 2). IL-1β mRNA expression has been reported to be increased 2 to 3.5-fold 1 to 3 days following injury in response to intense exercise in humans [59]–[61]. Data from our group revealed ∼100-fold increase in IL-1β mRNA 2 days after skeletal muscle injury using BaCl_2_ (data in press). The findings from the present study indicate that while IL-1β mRNA was not elevated 1 day after BaCl_2_ injury, there was a near 20-fold increase after 5 days (p≤0.05) (Figure 2A). These results, together with those of previous studies, indicate that peak IL-1β expression is likely to be between day 2 and 3 after injury, but remains elevated at least up to 5 days post-injury.

Similar to IL-1β, IL-6 mRNA has been shown to be elevated ranging from 2 hours to 3 days following injury (intense resistance and eccentric exercise) [61], [62]; however, it has also been reported that no increase was observed at 3 days following acute resistance exercise [60]. Our data demonstrate that IL-6 was not elevated at day 1 after muscle injury. However, there was a near 20-fold increase (p = 0.06) at 5 days following injury (Figure 2B). This elevated expression of IL-6 mRNA diminished back to baseline by day 10 following injury.

**Figure 3:**
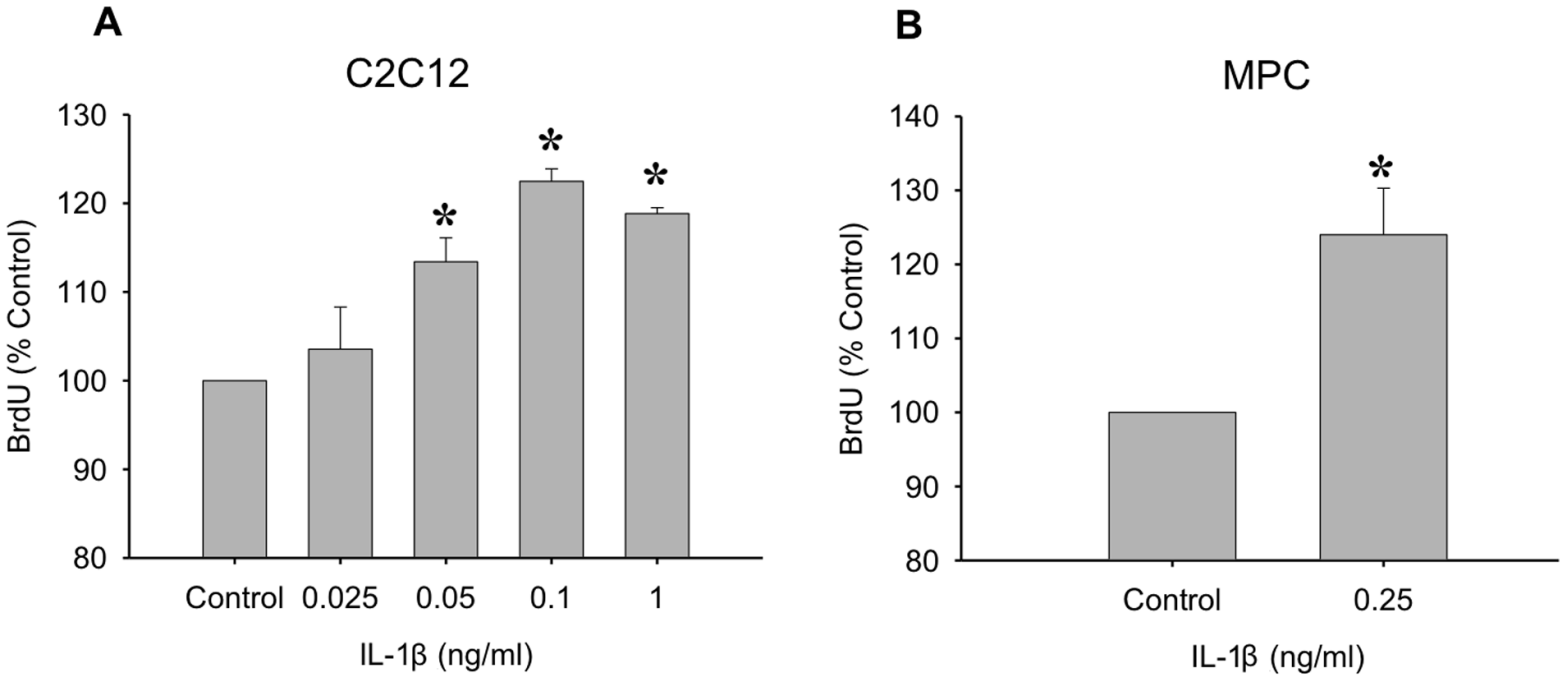
The mitogenic effects of IL-1β. (A) An IL-1β dose response was performed on C2C12 myoblasts. Concentrations of 0.5 ng/ml to 1 ng/ml significantly increased proliferation in myoblasts. (B) An intermediate dose of IL-1β (0.25 ng/ml) was used to test the mitogenic effects of IL-1β on primary muscle precursor cells. Data are expressed relative to control ± SEM. *denotes significance (p≤0.05) compared to control (n = 3–5 per dose).

**Figure 4:**
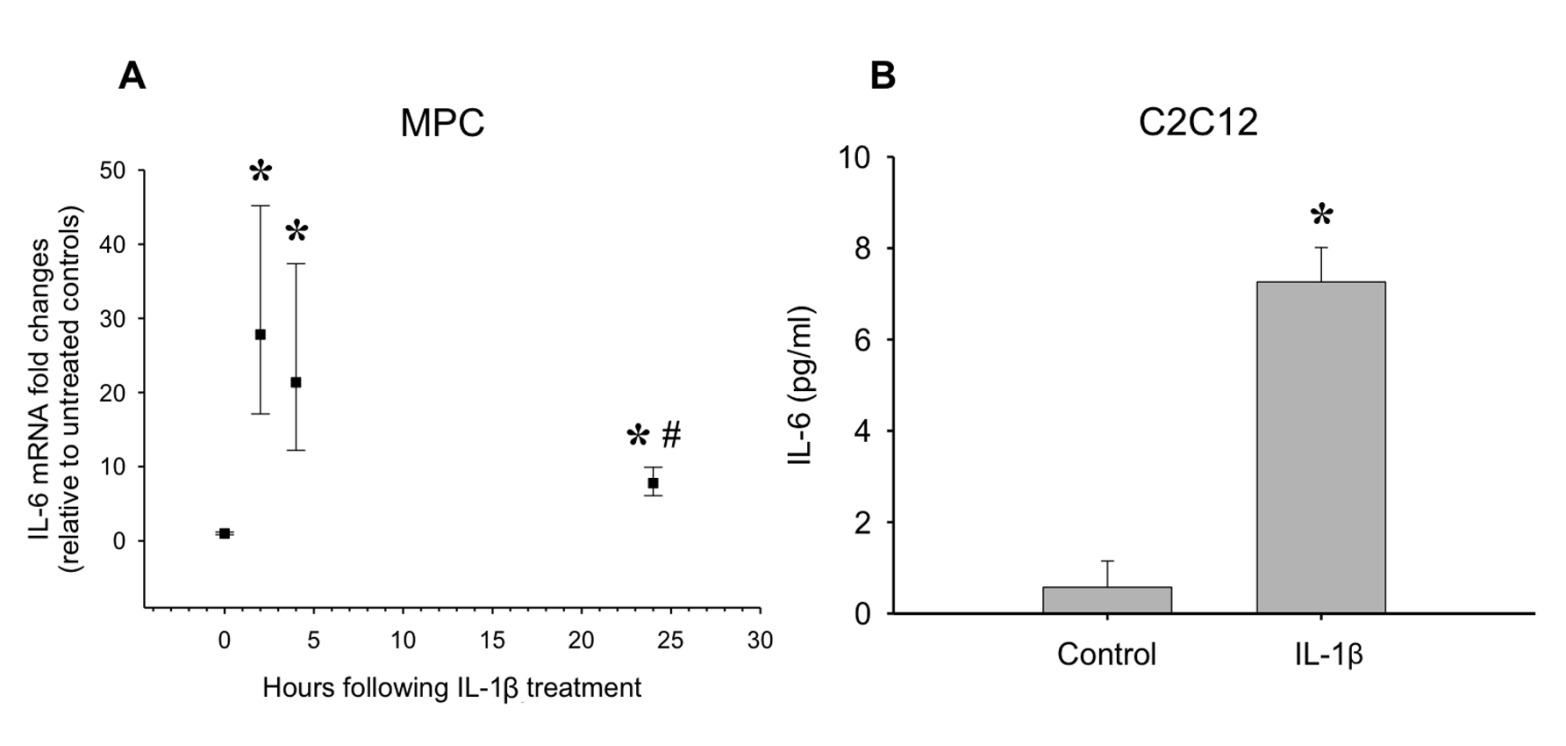
The effect of IL-1β treatment on IL-6 mRNA and protein levels. (A) Primary MPCs were treated with IL-1β (0.25 ng/ml) and IL-6 mRNA was determined over 24 hours. *denotes significance (p≤0.05) compared to control. # denotes significance (p≤0.05) compared to the 2 hour time point (n = 4 per time point). (B) IL-1β treatment (1 ng/ml) also increased IL-6 protein released into the media in C2C12 myoblasts. *denotes significance (p≤0.05) compared to control (n = 4–6).

Since it was shown that IL-1β expression is high during early regeneration, which is a critical time for MPC proliferation, we determined the direct effects of IL-1β treatment on proliferation (Figure 3). A dose response in the C2C12 myoblast cell line revealed that concentrations of IL-1β from 0.05 ng/ml to 1 ng/ml caused a significant increase in proliferation (p≤0.05; Figure 3A). An intermediate dose was chosen to test on primary MPCs and a ∼24% increase in proliferation was observed (p≤0.05; Figure 3B). Along with proliferative effects, IL-1β has also been shown to induce IL-6 expression [63], [64], which we tested in MPCs and myoblasts. In primary MPCs, IL-1β caused an increase in IL-6 mRNA expression as early as 2 hours post-stimulation (Figure 4A). The elevated IL-6 mRNA levels declined by 24 hours post-stimulation, but still remained elevated compared to control (p≤0.05; Figure 4A). In addition, C2C12 myoblasts treated with 1 ng/ml IL-1β for 24 hours released IL-6 protein into the media (p≤0.05; Figure 4B).

**Figure 5:**
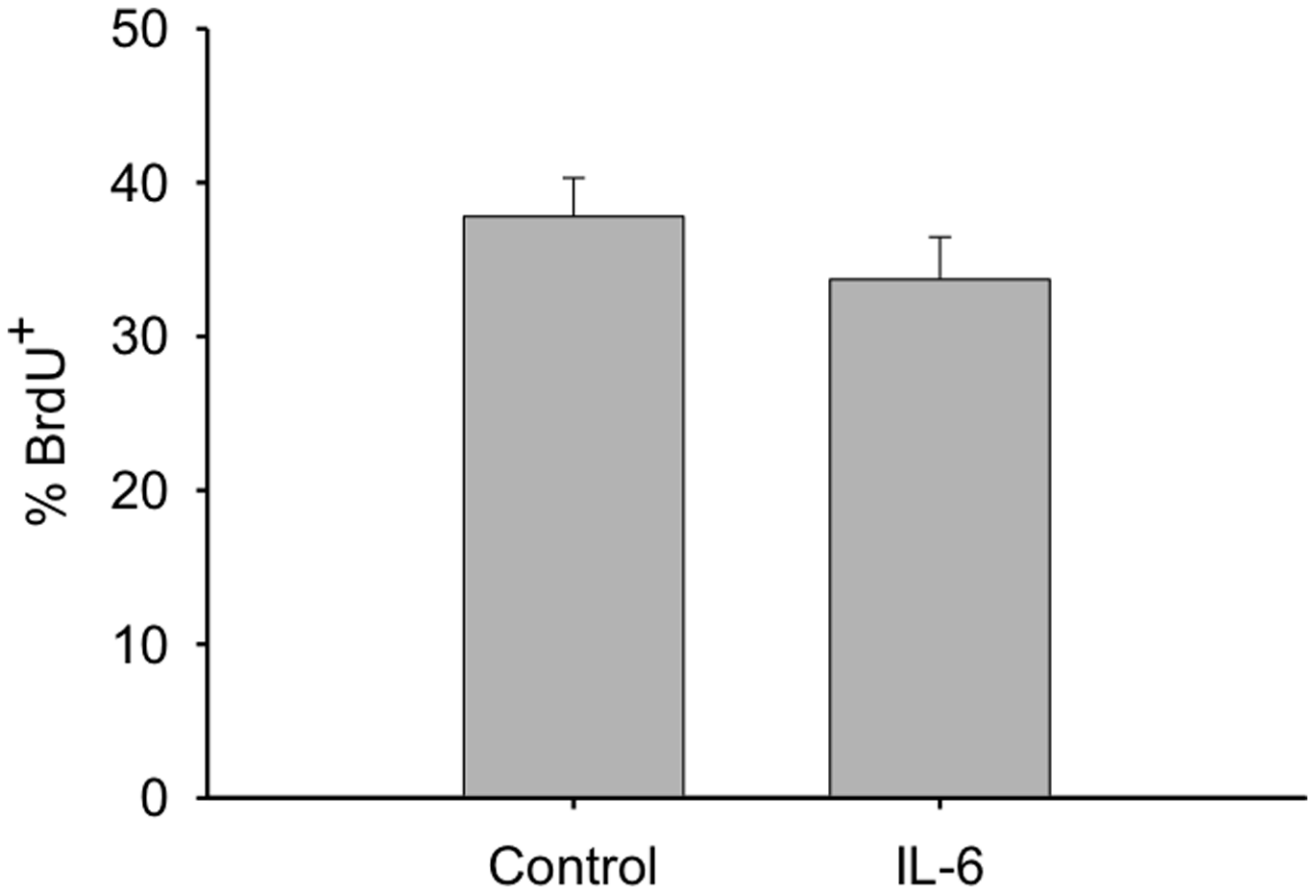
The mitogenic effects of IL-6 on C2C12 myoblasts. Myoblasts were treated with IL-6 (1 ng/ml) and BrdU incorporation was determined 24 hours after treatment (n = 5).

Since IL-6 is known to possess mitogenic effects on MPCs [31], [32], we tested whether it was possible that the increased proliferation induced by IL-1β could be a result of the IL-6 released by the myoblasts. To do this the mitogenic effects of IL-6 on C2C12 myoblasts were tested however, incubation with IL-6 for 24 hours did not alter BrdU (Figure 5). Therefore, the mitogenic effect of IL-1β does not appear to act through IL-6 expression in myoblasts.

**Figure 6:**
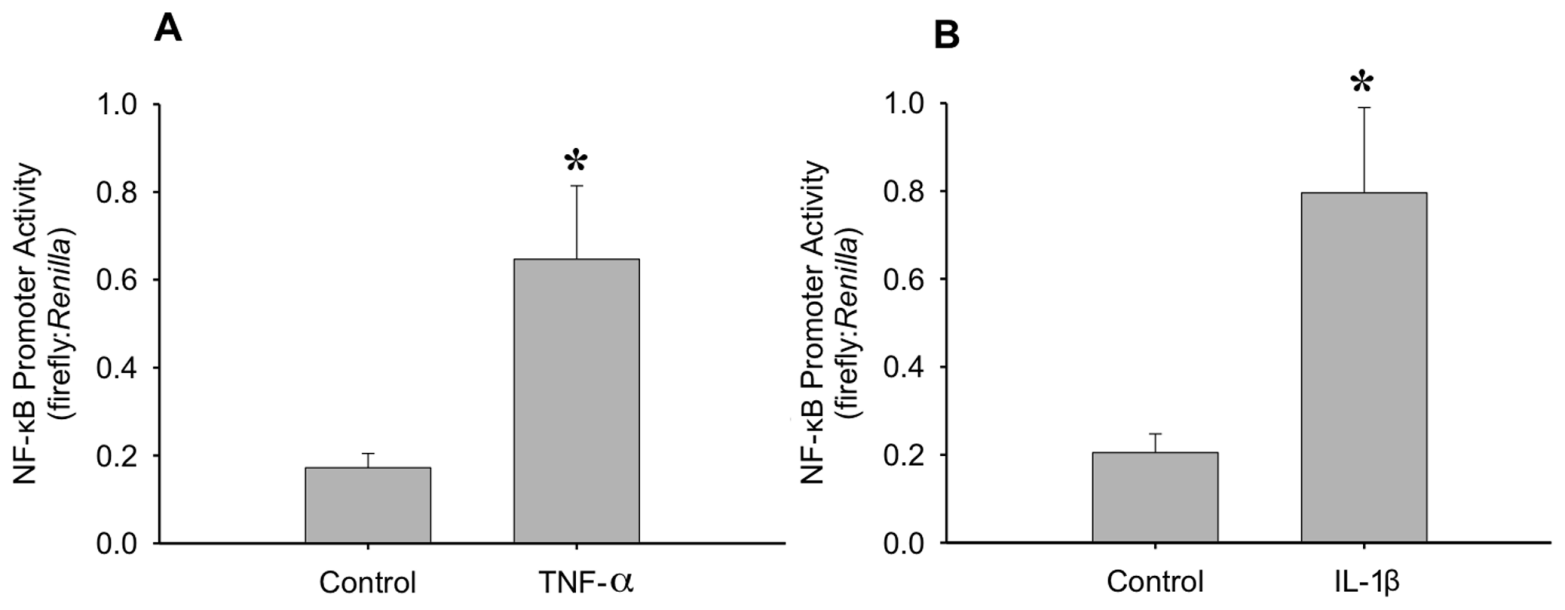
The effects of TNF-α and IL-1β on nuclear factor-kappa B (NF-κB) activity. Transfection of myoblasts with NF-κB cis-reporter construct allowed the study of the effects of (A) TNF-α (20 ng/ml) and (B) IL-1β (1 ng/ml) on NF-κB activity. 24 hours after transfection, cells were treated with either TNF-α or IL-1β for an additional 24 hours. Data are reported as the ratio of firefly to *Renilla* luminescence *denotes significance (p≤0.05) compared to control (n = 7 for TNF-α and n = 6 for IL-1β).

**Figure 7:**
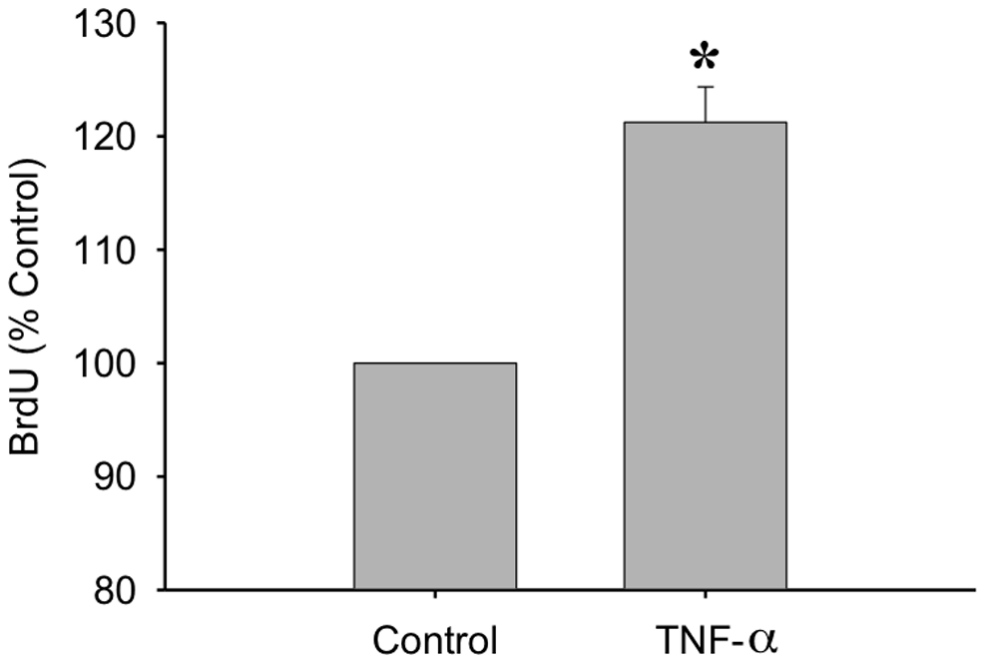
TNF-α promotes proliferation of myoblasts. BrdU incorporation was measured after a 24 hour TNF-α treatment (20 ng/ml). Data are expressed relative to control ± SEM. *denotes significance (p≤0.05) compared to control (n = 7).

It has also been shown that IL-1β expression can increase TNF-α expression [65] and this may occur through an NF-κB response element on the TNF-α promoter [66]. This increased TNF-α expression can also promote further activation of NF-κB [67]. In order to examine this sequence of events, we tested the effect of both IL-1β and TNF-α on NF-κB promoter activity as a measure of NF-κB activation (Figure 6). It was found that both TNF-α (Figure 6A) and IL-1β (Figure 6B) significantly increased NF-κB promoter activity, suggesting that IL-1β may be acting through TNF-α expression. It has been shown previously that TNF-α increases MPC proliferation [43] and this was confirmed in myoblasts when a ∼20% increase in BrdU incorporation was observed following treatment with 0.25 ng/ml TNF-α (Figure 7).

**Figure 8:**
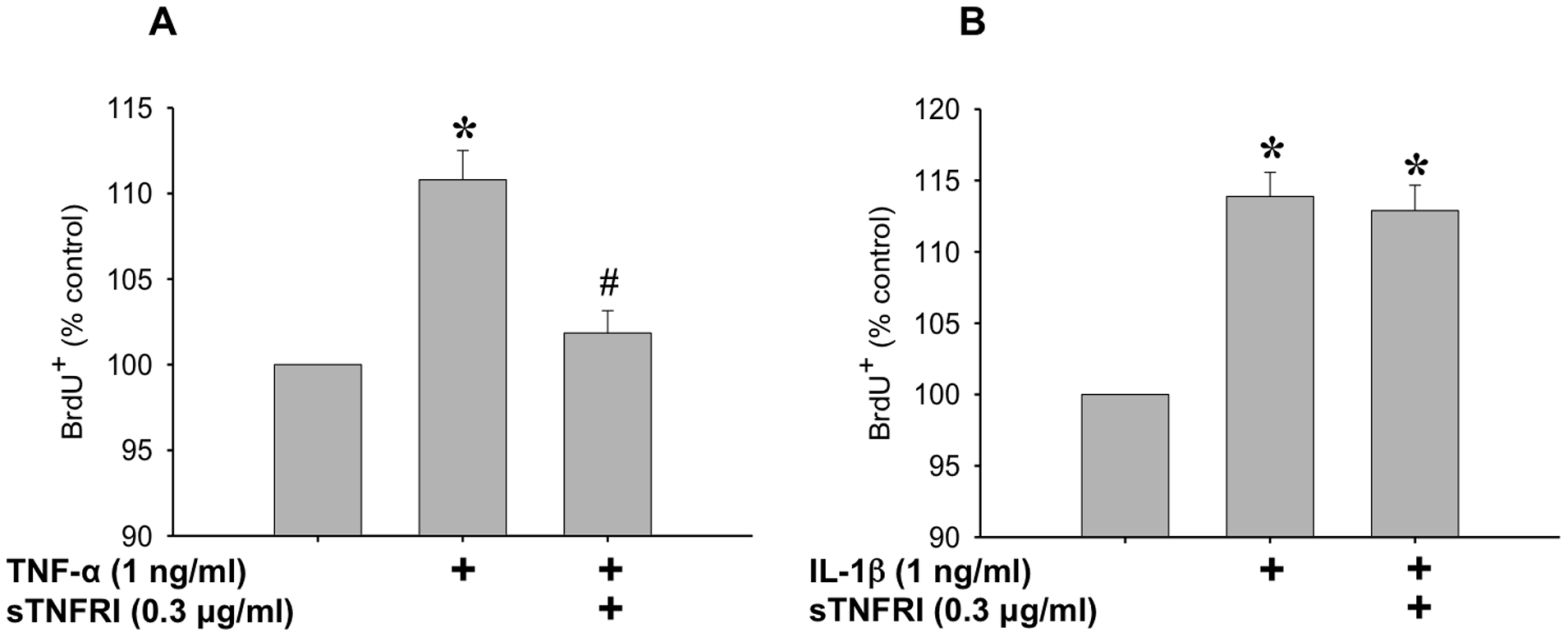
Determining the role of TNF-α in IL-1β mediated proliferation. (A) The effect of TNF-α on myoblast proliferation is blocked by pre-incubation of TNF-α with soluble TNF receptor I (sTNFRI) (0.3 μg/ml, 2 hours at 37°C). (B) IL-1β induced proliferation of myoblasts is not blocked by pre-incubation with sTNFRI. Data are expressed relative to control ± SEM. *denotes significance (p≤0.05) compared to control. # denotes significance (p<0.05) compared to TNF-α treated.

Since TNF-α expression was shown to increase proliferation, and has been shown to be induced by IL-1β expression [65], we examined whether IL-1β induced proliferation worked directly through TNF-α. To do this, BrdU incorporation was measured after myoblasts were treated with TNF-α or IL-1β, with and without pre-incubation with sTNFRI (Figure 8). A concentration of 0.3 μg/ml sTNFRI successfully inhibited TNF-α induced proliferation of myoblasts (Figure 8A). However, sTNFRI did not block IL-1β induced myoblast proliferation, suggesting that IL-1β induced proliferation may not work directly through TNF-α (Figure 8B).

**Figure 9:**
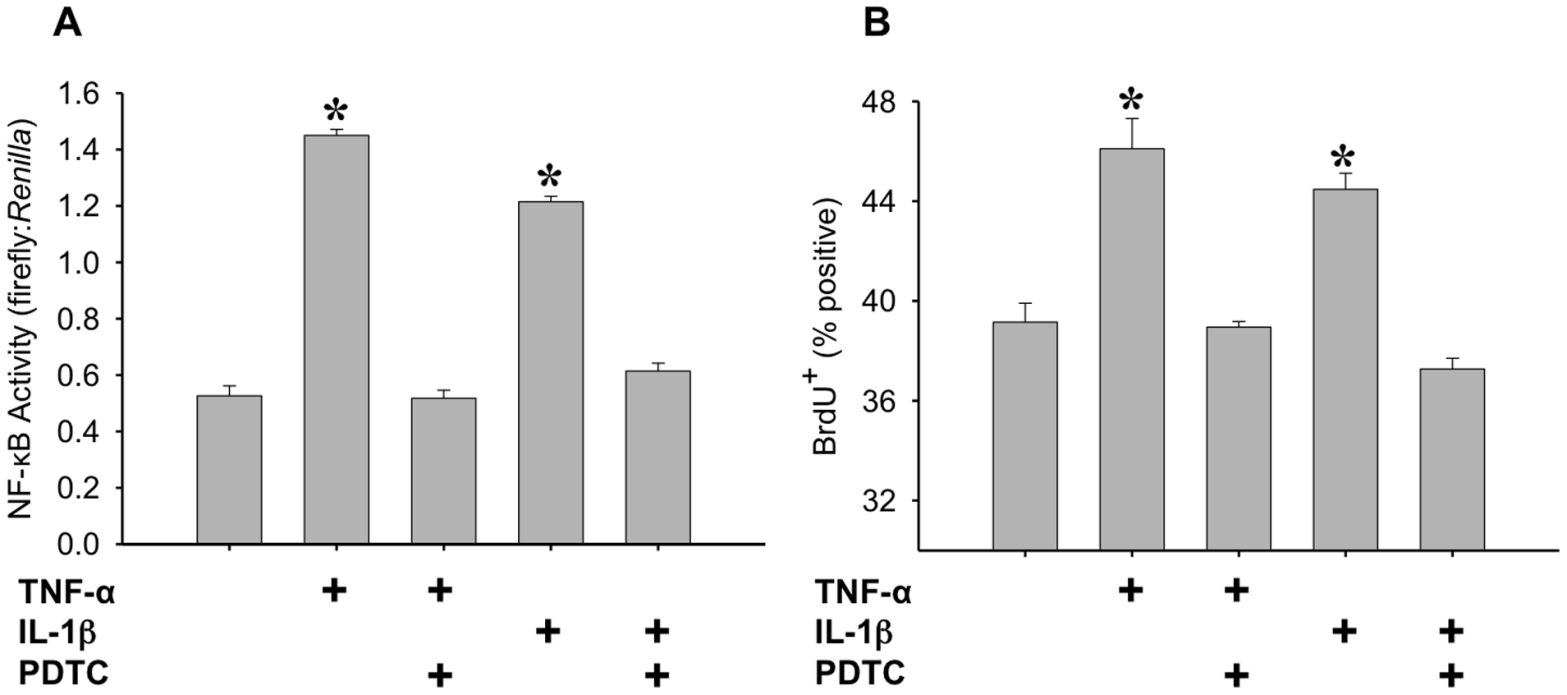
Determining the role of NF-κB activation in IL-1β and TNF-α induced proliferation. (A) NF-κB activity was determined using the NF-κB cis-reporter construct and data are reported as the ratio of firefly to *Renilla* luminescence. C2C12 myoblasts were transfected with the NF-κB cis-reporter construct, pretreated with 50 μM PDTC for 1.5 hours, and treated with either 1 ng/ml IL-1β or 20 ng/ml TNF-α for four hours. *denotes significance (p≤0.05) compared to control (n = 4). (B) Proliferation of myoblasts was measured through BrdU incorporation in C2C12 myoblasts following a 50 μM PDTC pretreatment for 1.5 hours, and a 20 hour treatment with either 1 ng/ml IL-1β or 20 ng/ml TNF-α. *denotes significance (p≤0.05) compared to control (n = 4).

In order to test whether IL-1β and TNF-α induced proliferation were dependent on the activation of NF-κB, we used pyrrolidine dithiocarbamate (PDTC) to inhibit NF-κB. PDTC has been shown to be effective at decreasing NF-κB activation in C2C12 myotubes [68]–[71] and myoblasts [72] at concentrations ranging from 10 μm to 100 μM. In order to observe this effect in proliferating C2C12 myoblasts, NF-κB promoter activity and BrdU incorporation were measured following treatment with IL-1β or TNF-α, with and without 50 μM PDTC. This concentration successfully inhibited NF-κB activation, and consequently, blocked proliferation induced by IL-1β or TNF-α (Figure 9 A and B). These data demonstrate that both IL-1β and TNF-α work to induce proliferation through NF-κB activation.

## Discussion

The early inflammatory cascade in skeletal muscle injury involves the secretion of pro-inflammatory cytokines by infiltrating immune cells [9]–[13]. Neutrophil infiltration can occur within 24 hours of injury, followed by subsequent macrophage and T cell accumulation that can persist for 10–14 days [52]. Previous reports have focussed on increased cytokine expression within the first 3 days following injury [73]. In the present study, we extended our knowledge of IL-1β and IL-6 expression over 28 days following injury. We found that IL-1β mRNA begins to increase as early as 1 day following injury and is significantly elevated ∼20-fold 5 days post-injury. IL-6 mRNA does not appear to increase above baseline 1 day following injury but is also ∼20-fold higher 5 days post-injury. Satellite cell/MPC proliferation occurs coincident with increased IL-1β and IL-6 expression and to our knowledge, we are the first to report that IL-1β increases proliferation of primary MPCs. Moreover, we provide further evidence of IL-1β/NF-κB signalling that is likely involved in the regulation of MPCs following injury.

While IL-1β has been shown to promote proliferation in other cell types [74], [75], less is known about its mitogenic role in response to skeletal muscle injury. Following a contusion injury, the concentrations of IL-1β in rat tibialis anterior muscle were found to be ∼200 pg/mg muscle protein [76]. Once water content of muscle and the fact that IL-1β occupies the interstitial space are accounted for, the conversion would be roughly ∼7 ng/ml in the interstitial space. In our study, we first tested a dose response of IL-1β ranging from 0.05 to 1.0 ng/ml on BrdU incorporation in C2C12 myoblasts. Our doses are within what was reported by Almeida et al. [76] and therefore represent concentrations that are likely achievable in response to injury. We found that a maximal response was observed at 0.1 and 1.0 ng/ml IL-1β. In addition, when primary MPCs were treated with an intermediate dose (0.25 ng/ml), a ∼25% increase in BrdU incorporation was observed. To our knowledge, this is the first report of IL-1β treatment alone increasing primary MPC proliferation. These findings are significant because the local milieu of injured skeletal muscle is dictated by immune cell derived cytokines and for MPCs to effectively contribute to muscle repair, they must respond and thrive in the presence of high concentrations of pro-inflammatory cytokines.

Previous reports have found that IL-6 expression is elevated 1 to 3 days following injury [61], [62]. In the present study, we found that IL-6 mRNA may remain elevated, although not significantly, ∼20-fold 5 days post-injury. Importantly, we also found that IL-1β increased IL-6 mRNA and protein release from primary MPCs and C2C12. It has been shown that IL-1β can regulate IL-6 production in various cell types including smooth muscle cells, enterocytes, endothelial cells, and fibroblasts [77]–[82]. Furthermore, IL-1β can increase IL-6 expression and release in C2C12 skeletal muscle myoblasts [63], [64] and C2C12 myotubes [35]. To our knowledge, this is the first report demonstrating IL-1β induces endogenous IL-6 expression in primary MPCs, which has implications for mitogenic effects *in vivo*. Our data support the notion that primary MPCs are a significant source of IL-6 in response to skeletal muscle injury.

IL-6 has been shown to have mitogenic effects on MPCs, and therefore, may play a key role in the regulation of skeletal muscle cell proliferation and regeneration [31], [83], [84]. IL-6^−/−^ mice displayed impaired regeneration, decreased macrophage infiltration, and inhibited myoblast proliferation [33]. While IL-6 has been shown to promote proliferation of C2C12 myoblasts [31], this was only observed at a concentration of 10 ng/ml. IL-6 at 1 ng/ml, the same dose used in the present study, did not affect proliferation [31]. Interstitial IL-6 concentration in the local milieu of MPCs in the injured skeletal muscle compartment is not currently known. We chose to test IL-6 at 1 ng/ml, a dose well above what was observed in the cell culture media after IL-1β treatement (Figure 4B). Since IL-6 did not increase proliferation at 1 ng/ml in the present study, it is not likely that the observed increase in proliferation in response to IL-1β stimulation is a result of endogenous IL-6 release.

Along with increasing IL-6 release, stimulation with 1 ng/ml IL-1β increased the activation of NF-κB to a similar extent as TNF-α at a concentration of 20 ng/ml. IL-1β has been shown to increase TNF-α expression and release in several cell types and tissues [65], [85]–[88], and we demonstrated that TNF-α increases proliferation of myoblasts; therefore we investigated the role of TNF-α in IL-1β induced proliferation. If IL-1β induced proliferation were a result of increased TNF-α release from the myoblasts, then one would expect that blocking TNF-α using the sTNFRI would prevent IL-1β induced proliferation. However, while sTNFRI was able to prevent TNF-α induced proliferation, there was no attenuation of IL-1β effects. Even though IL-1β does increase NF-κB activity, leading to increased proliferation, it does so independently of endogenous TNF-α expression. IL-1β and TNF-α have a common signalling protein termed NF-κB-inducing kinase [89]. Both cytokines may increase expression and activation of NF-κB through their respective receptors [66], [89]–[92]. In addition, IL-1β and TNF-α have both been shown to act via p38 MAPK [93]–[96]. Baeza-Raja *et al.* [97] reported cross-talk between the p38 MAPK and NF-κB pathways leading to NF-κB activation and increased myogenic progression [97], therefore p38 MAPK may also be a common signalling molecule for both cytokines.

In order to confirm the direct effect of each cytokine on NF-κB activation, and also the importance of this activation in IL-1β and TNF-α induced proliferation, PDTC was used to block NF-κB. PDTC is a known inhibitor of NF-κB in many cell types [70], [72], [98], [99], and Hayakawa *et al.* [98] discovered that it works through blocking the polyubiquitylation of IκB. In the present study, a concentration of 50 μM PDTC completely inhibited cytokine-induced NF-κB activity, resulting in decreased myoblast proliferation. In terms of skeletal muscle injury, IL-1β is likely one of many candidates that could increase NF-κB activity leading to a mitogenic response on MPCs. Along with TNF-α, which is elevated after injury [58] and increases proliferation (Figure 7), reactive oxygen species production is also increased after injury leading to increased NF-κB activation [100].

The timeline of expression of IL-1β and IL-6 coincides with activation and proliferation of MPCs, therefore the present study improved our understanding of the role of pro-inflammatory cytokines in skeletal muscle regeneration. We found that IL-1β does have a mitogenic effect in both primary MPCs and C2C12 myoblasts. Also, an IL-1β/TNF-α/IL-6 axis may exist, in that we report that IL-1β can increase the expression of IL-6, while previous research has shown that TNF-α may also increase IL-6. Importantly, both IL-1β and TNF-α increase NF-κB activation and proliferation of myoblasts. It seems likely that this common signalling through NF-κB is related to the mitogenic effect observed. While there is a common NF-κB activation, the IL-1β induced proliferation is not dependent on IL-1β stimulated TNF-α expression. These findings are important for highlighting the existing knowledge gaps regarding the pro-inflammatory milieu following skeletal muscle injury, and the synergistic relationship between the inflammatory response and the mechanisms regulating MPC function. Advancing our understanding of this relationship will facilitate the development of new treatments aimed at improving skeletal muscle regeneration in aging and disease.
